# Virulence Factors Contributing to Pathogenicity of *Candida tropicalis* and Its Antifungal Susceptibility Profile

**DOI:** 10.1155/2014/456878

**Published:** 2014-04-02

**Authors:** Sachin C. Deorukhkar, Santosh Saini, Stephen Mathew

**Affiliations:** Department of Microbiology, Rural Medical College, Loni, Maharashtra 413736, India

## Abstract

The incidence of invasive candidiasis has increased over the past few decades. Although *Candida albicans* remains by far the most common species encountered, in recent years shift towards non-*albicans Candida* species like *Candida tropicalis* is noted. Here in this study we determined the virulence factors and antifungal susceptibility profile of 125 *C. tropicalis* isolated from various clinical specimens. Biofilm formation was seen in 53 (42.4%) isolates. Coagulase production was noted in 18 (14.4%) isolates. Phospholipase enzyme was the major virulent factor produced by *C. tropicalis* isolates. A total of 39 biofilm forming isolates showed phospholipase activity. Proteinase activity was demonstrated by 65 (52%) isolates. A total of 38 (30.4%) isolates showed haemolytic activity. Maximum isolates demonstrated resistance to fluconazole. Fluconazole resistance was more common in *C. tropicalis* isolated from blood cultures. Antifungal resistance was more in isolates possessing the ability to produce phospholipase and biofilm. *C. tropicalis* exhibit a great degree of variation not only in their pathogenicity but also in their antifungal susceptibility profile. The identification of virulence attributes specific for each species and their correlation with each other will aid in the understanding of the pathogenesis of infection.

## 1. Introduction

Over the last three decades,* Candida* species has emerged as an important cause of health care associated and opportunistic infections [1]. The increased use of intravenous catheters, total parenteral nutrition, broad spectrum antibiotics, and cytotoxic chemotherapy and an increase in the population of immunocompromised patients have contributed to the increase of these infections [2].

Expression of virulence factors like germ tube formation, adhesins, phenotypic switching, thigmotropism, and biofilm formation and the production of hydrolytic enzymes contribute to the pathogenesis of candidiasis [1]. The clinical spectrum of candidiasis ranges from mucocutaneous overgrowth to disseminated infections like candidemia [3]. Although most infections are attributed to* C. albicans*, the shift towards treatment resistant non-*albicans Candida* (NAC) species is evident in recent years [4, 5]. The problem of emergence of NAC spp. becomes more acute because different species of NAC exhibit varying degrees of resistance, either intrinsic or acquired or both, to commonly used antifungal drugs.

*C. tropicalis* is one of the most common NAC spp. isolated from various clinical types of candidiasis [6]. In India,* C. tropicalis* is the most common cause of health care associated candidemia [7]. The increased isolation of* C. tropicalis* from various clinical types of candidiasis is of concern because of its ability to develop rapid resistance to fluconazole [8].

Among* Candida* spp., expression of virulence factors may vary depending on the infecting species, geographical origin, type of infection, the site and stage of infection, and host reaction. Knowledge of these virulence factors will be an important tool to understand pathogenesis of candidiasis and in addition will help explore new antifungal drug targets for improved therapeutic regimens. A review of the available literature has revealed a dearth of information regarding the epidemiology, pathogenesis, virulence factors, and antifungal susceptibility patterns of* C. tropicalis*. Therefore the present study was taken up with an aim to study the virulence factors and antifungal susceptibility profile of* C. tropicalis* isolated from various clinical specimens.

## 2. Materials and Methods

The present study was conducted in the Department of Microbiology, Rural Medical College and Hospital of Pravara Institute of Medical Sciences, Loni, Maharashtra, and is part of a PhD thesis. The protocol of the study was approved by the institutional ethics committee (Registration no. PIMS/PhD/RC/2013/24).

A total of 125* C. tropicalis* isolated from various clinical samples were included in the study.* C. tropicalis* was identified by HiCandida identification kit and colony color on Hichrome* Candida* agar (Himedia Laboratories Pvt. Ltd., Mumbai, India).

The virulence factors studied were exoenzymatic activity (coagulase, phospholipase, and proteinase), biofilm formation, and haemolysin production.

### 2.1. Coagulase Activity

Coagulase production by* C. tropicalis* was detected by the method of Yigit et al. [9]. Approximately 0.1 mL of an overnight culture of* C. tropicalis* was aseptically inoculated into a tube containing 500 *μ*L of rabbit plasma. The tubes were incubated at 35°C and observed for clot formation after 2, 4, 6, and 24 h.

The presence of a clot that could not be resuspended by gentle shaking indicated positive coagulase test.* Staphylococcus aureus* ATCC 25923 and* S. epidermidis* ATCC 14990 were used as positive and negative controls, respectively.

### 2.2. Phospholipase Production

The phospholipase activity of* C. tropicalis* was detected by the method of Samaranayake et al. [10]. Approximately 5 *μ*L of standard inoculum of test strain containing 10^8^ *Candida* cells/mL was aseptically inoculated onto egg yolk agar. The plates were dried at room temperature and then incubated at 37°C for 48 h. The plates were examined for the presence of precipitation zone around the colony. The presence of precipitation zone indicated expression of phospholipase enzyme.* C. albicans* ATCC 10231 was used as positive control.

The phospholipase index (Pz) was defined as the ratio of the diameter of the colony to the total diameter of the colony plus the precipitation zone. A Pz value of 1 denoted no phospholipase activity; Pz < 1 indicated phospholipase production by the isolate. The lower the Pz value, the higher the phospholipase activity [11]. To minimize experimental error, the assay was conducted in duplicate on three separate occasions for each isolate.

### 2.3. Proteinase Activity

Proteinase activity of* C. tropicalis* isolates was screened by the method described by Staib [12]. It was measured in terms of bovine serum albumin (BSA) degradation. Approximately 10 *μ*L of standard inoculum (10^6^ *Candida* cells/mL) was aseptically inoculated on to 1% BSA plate. The plate was incubated for 5 days at 37°C.* C. albicans* ATCC 10231 was used as positive control.

After incubation, further proteinase activity was inhibited by adding 20% trichloroacetic acid and the plate was stained with 1.25% amidoblack. A zone of proteolysis surrounding the colony that could not be stained with amidoblack indicated proteinase activity.

The proteinase index (Prz) was measured in terms of the ratio of the colony to the diameter of unstained zone. A Prz value of 1 indicated no proteinase activity; Prz < 1 denoted proteinase expression by the isolate. The lower the Prz value, the higher the proteinase activity [11]. To minimize experimental error, the assay was conducted in duplicate on three separate occasions for each isolate.

### 2.4. Haemolysin Production

Haemolytic activity of* C. tropicalis* was screened on sheep blood Sabouraud dextrose agar plate by the method described by Manns et al. [13]. Approximately 10 *μ*L of standard inoculum (10^8^ *Candida* cells/mL) was aseptically inoculated onto the medium. The culture plates were incubated at 37°C for 48 h.* C. albicans* ATCC 90028 was used as the control strain.* Streptococcus pyogenes* (Lancefield group A) and* Streptococcus sanguis* were used as positive controls for beta and alpha haemolysis, respectively.

The presence of a zone of haemolysis around the colony indicated haemolysin production. Haemolytic activity (Hz) was calculated in terms of the ratio of diameter of the colony to that of the translucent zone of haemolysis (in mm).

### 2.5. Biofilm Formation

The ability of* C. tropicalis* isolates to form biofilms was assessed by the tube method described by Yigit et al. [9]. Colonies of* C. tropicalis* from Sabouraud dextrose agar were inoculated in saline and incubated overnight at 37°C. 0.5 mL of this saline suspension was added into screw capped conical polystyrene tubes containing 5 mL of Sabouraud dextrose broth supplemented with glucose (final concentration of 8%). The tubes were incubated at 35°C for 48 h without agitation.

After incubation the broth from the tubes was aspirated gently using Pasteur pipette. The tubes were washed twice with distilled water and stained with 2% safranin. The stain was decanted after 10 min. The tubes were rinsed with distilled water to remove excess stain.

Presence of visible adherent film on the wall and at the bottom of the tube indicated biofilm formation. Ring formation at the liquid interface was not considered as an indication of biofilm production [11].* Staphylococcus epidermidis* ATCC 35984 and* C. albicans* ATCC 10231 were used as positive and negative controls, respectively.

### 2.6. Antifungal Susceptibility Testing

The antifungal susceptibility testing of* C. tropicalis* isolates was performed using Hicomb minimum inhibitory concentration (MIC) test (Himedia Laboratories Pvt. Ltd., Mumbai, India). The antifungal agents tested were amphotericin B (range 0.002–32 *μ*g), fluconazole (range 0.016–256 *μ*g), itraconazole (range 0.002–32 *μ*g), and ketoconazole (range 0.002–32 *μ*g).

The inoculum was prepared by inoculating 3-4 colonies of the* C. tropicalis* isolate to be tested in saline. The turbidity of suspension was matched with 0.5 McFarland standard. The suspension was inoculated on the agar plate containing RPMI 1640 supplemented with 2% glucose by lawn culture method using tipped cotton swab. The manufacturer's instructions were adhered to throughout the test. The antifungal strips were aseptically placed on the media with the help of forceps and the plates were incubated at 35°C for 24–48 h.* C. albicans* ATCC 90028 and* C. parapsilosis* ATCC 22019 were used for quality control.

The results of antifungal susceptibility test were interpreted as sensitive (*S*), dose-dependent susceptible (DDS), and resistant (*R*). Interpretative criteria for azoles were those recommended by the Clinical Laboratory Standard Institute (CLSI) [14, 15]. Due to the lack of defined breakpoints for amphotericin B arbitrary values based on the studies of other researchers were used [11, 16].

## 3. Results

**Figure 1:**
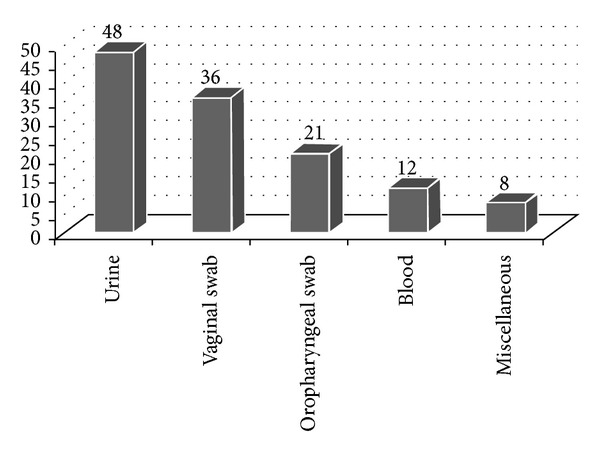
Sample-wise distribution of* Candida tropicalis*.

Figure 1 shows the clinical specimen wise distribution of* C. tropicalis*. Majority of the isolates were obtained from urine samples (38.4%) followed by vaginal swabs (28.8%). Indwelling urinary catheters, use of antibiotics, geriatric patients, and diabetes mellitus were risk factors found associated with* C. tropicalis* UTI. Pregnancy, uncontrolled diabetes, and use of low dosage azole maintenance regimen were predisposing factors for* C. tropicalis* vulvovaginitis. HIV was the major predisposing factor for oropharyngeal candidiasis (OPC). ICU stay, total parenteral nutrition (TPN), prior exposure to fluconazole, and diabetes mellitus were the major risk factors for candidemia.

Virulence factors produced by* C. tropicalis* isolates are shown in Table 1. Biofilm formation was seen in 53 (42.4%) isolates.* C. tropicalis* isolated from urine and blood samples demonstrated high biofilm production capacity. Coagulase production was noted in 18 (14.4%) isolates. Of these, only 2 isolates showed coagulase expression within 4 h of incubation. Coagulase production was more in blood isolates. Phospholipase enzyme was the major virulent factor produced by* C. tropicalis* isolates.* C. tropicalis* isolated from vaginal swabs and blood cultures showed maximum phospholipase activity. A total of 39 biofilm forming isolates showed phospholipase activity. Proteinase activity was demonstrated by 65 (52%) isolates. Proteinase production was high in* C. tropicalis* isolated from vaginal swabs and oropharyngeal swabs. A total of 38 (30.4%) isolates showed haemolytic activity. All isolates showed *β* type of haemolysis.

Antifungal susceptibility profile of* C. tropicalis* isolates is shown in Table 2. Maximum isolates demonstrated resistance to fluconazole (71.2%) followed by ketoconazole (68%). Fluconazole resistance was more common in isolates obtained from cases of candidemia, OPC, and vulvovaginal candidiasis. Amphotericin B resistance was seen in 22 isolates. It was more common in* C. tropicalis* isolated from blood cultures and oropharyngeal swabs. Antifungal resistance was more in isolates possessing the ability to produce phospholipase and biofilm.

Miscellaneous samples included Foley's catheter tips, ear swab, endotracheal tube, and pleural fluid.

## 4. Discussion

Mycosis in general and candidiasis in particular are both widespread and increasing in frequency. The increased frequency of* Candida* infections to a certain extent coincides with advances in the field of medicine [17]. NAC spp. once dismissed or ignored as nonpathogenic, commensal, or contaminant have emerged as potential pathogens [18]. Among NAC spp.* C. tropicalis* alone, or in association with other species, is implicated more frequently in human infections [19].

In this study,* C. tropicalis* was most commonly isolated from urine samples. Paul et al. [20] reported* C. tropicalis* as the most prevalent NAC spp. causing candiduria. In the study by Jain et al. [21],* C. tropicalis* was the predominant cause of candiduria in catheterized ICU patients. The major risk factors for candiduria included indwelling catheters, recent use of antibiotics, advanced age, and diabetes mellitus. Indwelling urinary catheters facilitates the entry and colonization of* Candida *[22]. Use of broad spectrum antibiotics helps in colonization by* Candida* by suppressing normal bacterial flora of gut and lower genital tract [22]. Diabetes not only impairs host immunity but also increases* Candida* colonization by promoting stasis of urine in neurogenic bladder [21].

In recent years, many studies have shown an increased prevalence of VVC due to NAC spp. In our study, pregnancy, uncontrolled diabetes, and use of low dose azole maintenance regimen were major predisposing factors for* C. tropicalis* vulvovaginitis. Widespread and inappropriate use of antifungal therapy in the form of self-medication, long term maintenance dosage, and use of a single dose oral and topical azole results in eradication of* C. albicans* and selection of NAC spp. that are resistant to commonly used antifungal drugs [23].

OPC is the most common opportunistic mycoses in immunocompromised individuals. In our study, HIV infection was the most common predisposing factor for OPC. OPC occurs in up to 90% of HIV infected individuals during the course of infection [24]. In recent years NAC spp. like* C. tropicalis*,* C. glabrata,* and* C. krusei* have been increasingly recovered from HIV patients with OPC [25]. Predominance of* C. tropicalis* among NAC spp. as a causative agent of OPC in HIV infected individuals was also noted in studies by other researchers [16, 25].

Candidemia is an important complication in severely ill hospitalized patients [26]. The increased isolation rates of NAC spp. from blood stream infections along with a gradual shift in the antifungal susceptibility profile are documented in many recent studies [7]. In our study ICU stay, TPN, prior exposure to fluconazole, and diabetes mellitus were risk factors identified to be associated with* C. tropicalis* candidemia. The increased use of fluconazole is considered a major cause for increase of* C. tropicalis* candidemia [7]. Studies of various researchers from different parts of India have reported* C. tropicalis* to be the most prevalent NAC spp. isolated from candidemia cases [27, 28].

Extensive research on these virulence factors is focused on* C. albicans*, which is considered the most pathogenic member of the genus [1]. However, quite a few research articles refer to virulence factor production in NAC spp. In the present study, biofilm formation was noted in 42.4% of* C. tropicalis *isolates.* Candida* spp. possess ability to form biofilm on most, if not all, medical devices [29]. Singhai et al. [30] reported* Candida *associated catheter related sepsis in 7.4% of patients with peripheral intravascular catheters. Detection of biofilm forming ability in* Candida* spp. is of utmost importance as these organisms not only colonize medical devices, but also lead to resistant health care associated infections [1].

Extracellular hydrolytic enzymes play an important role in the pathogenesis of candidiasis [31]. These enzymes facilitate adaptation to distinct types of infection and enhance survival of the pathogen. Most of the studies on exoenzymes are focused on phospholipases and secreted aspartyl proteinases (Sap) [32]. Coagulase production and haemolytic activity of* C. tropicalis* are the least studied. Coagulase binds plasma fibrinogen and activates a cascade of reactions that induce clotting of plasma [33]. In our study coagulase production was seen in 14.4% of* C. tropicalis* isolates. Rodrigues et al. [33] reported high coagulase activity in* C. tropicalis* (82.6%). Haemolysin secretion followed by iron acquisition facilitates deeper tissue invasion by* Candida* [34]. In the present study, 30.4% of* C. tropicalis* showed haemolytic activity. Mane et al. [35] reported high haemolytic activity in* C. tropicalis* isolated from HIV infected individuals.

Among extracellular hydrolases, proteinases and phospholipases play major a role in host tissue invasion, colonization, and progression of infection [32]. Phospholipases facilitate the invasion of the host mucosal epithelia by hydrolyzing one or more ester linkages in glycerophospholipids [30]. In our study phospholipase was the major virulent factor expressed by* C. tropicalis* isolates. Researchers have reported contradictory findings regarding phospholipase activity in* C. tropicalis*. Investigators like Thangam et al. [36] reported high phospholipase activity in* C. tropicalis* isolates among NAC spp. while others like Samaranayake et al. [10] reported no activity. These inconsistencies in observations may be due to biological differences among the isolates tested. The present study also demonstrated high phospholipase activity in biofilm forming isolates. Screening of phospholipase production in biofilm forming isolates can be used as an important parameter to differentiate invasive strains from noninvasive colonizers.

Proteinases are capable of degrading host epithelial and mucosal barrier proteins such as collagen, keratin, and mucin. They also aid* Candida* to resist cellular and humoral immunity by degrading antibodies, complement, and cytokines [37]. A total of 52% of* C. tropicalis* isolates were proteinase producers. This observation was in agreement with other researchers like Deorukhkar and Saini [5], Mane et al. [35], and Dostá et al. [38].

Antifungal susceptibility testing is still less developed and utilized than antibacterial testing. The CLSI standardized broth microdilution method is complex and labor intensive to use as a routine method. Alternative methods like disc diffusion and Etest have been adapted for sensitivity testing of* Candida* spp. by resource limited hospital laboratories [39, 40].

In our study, resistance rates for the azole group of antifungal drugs were more as compared to amphotericin B. Azole resistance in* C. tropicalis* is insufficiently investigated [41]. In the present study,* C. tropicalis* isolates were found to be more resistant to fluconazole. Resistance to fluconazole is reported to have increased. Sanglard and Odds described overexpression of Ct*ERG11* gene associated missense mutation to be responsible for the acquired azole resistance in* C. tropicalis *[42].

The increase in the rate of fluconazole resistance in* C. tropicalis* is of concern because this species is one of the most commonly isolated NAC spp. and fluconazole is the most common antifungal agent used for the treatment of various types of candidiasis*. C. tropicalis* isolates from blood cultures demonstrated a higher rate of resistance to fluconazole. Our observation was in agreement with that of Yang et al. [8].

*C. tropicalis* capable of exhibiting certain virulence factors like biofilm formation and phospholipase production had higher rates of resistance to fluconazole. As compared to bacterial biofilms,* Candida* biofilms are resistant to many antimicrobial agents; the removal and replacement of infected medical device are required for effective treatment [1]. The exact mechanism of fluconazole resistance in phospholipase producing* Candida* spp. is not clearly understood [43]. More clinicomycological research is needed to explore this corelation.

## 5. Conclusion

Increased incidence of systemic candidiasis along with antifungal resistance has become an important healthcare issue worldwide. NAC spp. like* C. tropicalis* exhibit a great degree of variation not only in their pathogenicity but also in their antifungal susceptibility profile. The identification of virulence attributes specific for each species and their correlation with each other will aid in the understanding of the pathogenesis of infection. The importance of early and accurate identification of infecting* Candida* species along with susceptibility testing for timely institution of appropriate therapy cannot be overstated.

## Figures and Tables

**Table 1:** Production of various virulence factors by *Candida tropicalis*.

Virulence factor	Urine (*n* = 48)	Vaginal swab (*n* = 36)	Oropharyngeal swab (*n* = 21)	Blood (*n* = 12)	Miscellaneous (*n* = 12)	Total (*n* = 125)
Biofilm formation	32 (60.3%)	06 (11.3%)	04 (7.5%)	10 (18.8%)	01 (1.8%)	53
Coagulase production	05 (27.7%)	02 (11.1%)	02 (11.1%)	08 (44.4%)	01 (5.5%)	18
Haemolytic activity	06 (15.7%)	22 (57.8%)	03 (7.8%)	07 (18.4%)	—	38
Phospholipase activity	10 (13.8%)	34 (47.2%)	10 (13.8%)	11 (15.2%)	07 (9.7%)	72
Proteinase production	15 (23.1%)	25 (38.4%)	20 (30.7%)	04 (6.1%)	01 (1.5%)	65

**Table 2:** Antifungal susceptibility profile of *Candida tropicalis*.

Antifungal agent	Sensitive (%)	Dose-dependent sensitive (%)	Resistant (%)
Amphotericin B	96 (76.8)	07 (5.6)	22 (17.6)
Fluconazole	32 (25.6)	04 (3.2)	89 (71.2)
Itraconazole	35 (28)	06 (4.8)	84 (67.2)
Ketoconazole	36 (28.8)	04 (3.2)	85 (68%)
